# Das Lagemanagement des Robert Koch-Instituts während der COVID-19-Pandemie und der Austausch zwischen Bund und Ländern

**DOI:** 10.1007/s00103-021-03294-0

**Published:** 2021-03-05

**Authors:** Ariane Halm, Ulrike Grote, Maria an der Heiden, Osamah Hamouda, Lars Schaade, Ute Rexroth, RKI-Lagezentrums-Gruppe, Muna Abu Sin, Katharina Alpers, Iris Andernach, Esther-Maria Antao, Mona Askar, Jonathan Baum, Jan Baumann, Sandra Beermann, Jennifer Bender, Ides Boone, Matthias Borchert, Lena Bös, Sindy Böttcher, Michael Brandl, Viviane Bremer, Hellen Buijze, Luisa Denkel, Teresa Domaszewska, Sandra Dudareva, Julia Enkelmann, Werner Espelage, Sarah Esquevin, Mirko Faber, Christina Frank, Luam Ghebreghiorghis, Luise Goerlitz, Karin Gröschner, Barbara Gunsenheimer-Bartmeyer, Sebastian Haller, Nicole Hanschmann, Henriette Haub, Jane Hecht, Helena Heese, Wiebke Hellenbrand, Stefanie Herfort, Julia Hermes, Antonia Hilbig, Alexandra Hofmann, Alexandra Holzer, Claudia Houareau, Iris Hunger, Maren Imhoff, Jens Jacob, Klaus Jansen, Peter Kaiser, Katja Kajikhina, Basel Karo, Kerstin Kling, Judith Koch, Cyrus König, Uwe Koppe, Doreen Krause, Wiebe Külper, Isabella Kumpf, Raskit Lachmann, Franziska Layer, Ann-Sophie Lehfeld, Marina M. Lewandowsky, Nadine Litzba, Matthäus Lottes, Andrea Männel, Uli Marcus, Inessa Markus, Adine Marquis, Christian Mast, Sarah McFarland, Anika Meinen, Kai Michaelis, Felix Moek, Inge Mücke, Nadine Muller, Hildegard Niemann, Vanessa Ohm, Christiane Petasch, Kirsten Pörtner, Kerstin Prahm, Karina Preußel, Nadine Püschel, Sarah Reda, Sybille Rehmet, Felix Reichert, Lukas Reitzle, Anna Rohde, Eugenia Romo Ventura, Bettina Rosner, Andrea Sailer, Navina Sarma, Julia Schilling, Susi Schink, Christian Schmidt, Timm Schneider, Meike Schöll, Viktoria Schönfeld, Nina Schöpf, Madlen Schranz, Juliane Seidel, Janna Seifried, Regina Selb, Claudia Sievers, Regina Singer, Klaus Stark, Gyde Steffen, Daniel Stern, Anna Stoliaroff, Angelina Taylor, Kristin Tolksdorf, Sara Tomczyk, Petra von Berenberg, Anja von Laer, Robert Vonderwolke, Sabine Vygen-Bonnet, Jan Walter, Sabrina Weiß, Roland Wilhelm, Hendrik Wilking, Katja Winter, Veronika Wolf, Arina Zanuzdana, Nadine Zeitlmann, Ruth Zimmermann

**Affiliations:** 1 Abteilung für Infektionsepidemiologie, Robert Koch-Institut, Seestraße 10, 13353 Berlin, Deutschland; 2 Zentrum für Biologische Gefahren und Spezielle Pathogene (ZBS), Robert Koch-Institut, Berlin, Deutschland

**Keywords:** Lagezentrum, Robert Koch-Institut, Krisenmanagement, COVID-19, SARS-CoV‑2, Emergency operations centre, Germany, Crisis management, COVID-19, SARS-CoV‑2

## Abstract

Das Robert Koch-Institut (RKI) spielt in Deutschland eine zentrale Rolle bei der Bewältigung von Gesundheitsgefahren biologischen Ursprungs. Das Krisenmanagement des RKI hat das Ziel, dazu beizutragen, die Gesundheit der Menschen in Deutschland in epidemisch bedeutsamen Lagen zu schützen und die Arbeitsfähigkeit des RKI auch bei hoher Belastung über längere Zeit aufrechtzuerhalten. In diesem Artikel wird das Krisenmanagement des RKI generell und speziell während der COVID-19-Pandemie zum Stand 31.10.2020 dargestellt. Es werden die generischen RKI-Krisenmanagementstrukturen und der Aufbau des RKI-Lagezentrums, deren Operationalisierung im Rahmen der COVID-19-Pandemie sowie die aufgetretenen Herausforderungen beschrieben. Ebenso wird der Bund-Länder-Austausch während der bisherigen Pandemie dargestellt.

Die COVID-19-Pandemie hat außergewöhnliche Umstände mit sich gebracht. Eine gute Kommunikation und Koordination während der Lage sind essenziell, sowohl RKI-intern als auch mit anderen Bundes- oder Länderbehörden und der Fachöffentlichkeit. Unter hohem Druck erstellt das RKI Empfehlungen, Stellungnahmen und Bewertungen zu verschiedenen Themen und aktualisiert diese regelmäßig. Für die operative Unterstützung aller Aktivitäten wurde am RKI ein Lagezentrum aktiviert. Während der COVID-19-Pandemie gibt es verschiedene sowohl personelle als auch strukturelle Herausforderungen. Es zeigte sich, dass eine gute Vorbereitung (z. B. bereits vorhandene Positionsbeschreibungen und Räumlichkeiten) das Krisenmanagement positiv beeinflusst.

## Abstract (en)

The Robert Koch Institute (RKI) plays a central role in Germany in the management of health hazards of biological origin. The RKI’s crisis management aims to contribute to protecting the health of the population in Germany in significant epidemic situations and to maintain the RKI’s working ability over a long period of time even under high load. This article illustrates the crisis management of the RKI in general as well as during the COVID-19 pandemic. The generic RKI crisis management structures and the setup of the RKI emergency operations centre (EOC), their operationalisation in the context of the COVID-19 pandemic and the resulting challenges as of 31 October 2020 are described in this paper. The exchange between the federal and state governments during the pandemic is also described.

The COVID-19 pandemic has led to extraordinary circumstances. During the epidemic situation, good communication and coordination has been essential, both within the RKI and with other federal or state authorities and expert groups. Under great pressure, the RKI produces and regularly updates recommendations, statements and assessments on various topics. To provide operational support for all COVID-19 related activities, an EOC was activated at the RKI. During the COVID-19 pandemic, there are various challenges regarding personnel and structures. It became apparent that good preparation (e.g. existing task descriptions and premises) has an important positive impact on crisis management.

## Einleitung

Epidemisch bedeutsame Lagen sind in Art und Ausmaß nicht oder nur schwer vorhersehbar und können neue und unerwartete Formen annehmen. Sie erfordern eine hohe Flexibilität sowie eine schnelle Reaktion der verantwortlichen Institutionen. Krisenmanagement ist ein Kernbereich des öffentlichen Gesundheitswesens. Eine gute Vorbereitung auf gesundheitliche Notlagen kann die Effizienz der Reaktion in Krisenzeiten erheblich erhöhen und beschleunigen.

Das Robert Koch-Institut (RKI) ist die im Ressort des Bundesministeriums für Gesundheit (BMG) angesiedelte zentrale Forschungs- und Referenzeinrichtung sowohl für übertragbare als auch für nichtübertragbare Krankheiten der Menschen in Deutschland. Es ist für die Kontrolle und Prävention von Krankheiten sowie für die Herausgabe von Empfehlungen und Richtlinien für das öffentliche Gesundheitswesen und medizinische Fachkräfte verantwortlich [1]. Das RKI ist die Behörde, die im Rahmen der Internationalen Gesundheitsvorschriften (IGV) und des Beschlusses der Europäischen Union (EU) 1082/2013/EU für die Bewältigung von Gesundheitsgefahren biologischen Ursprungs zuständig ist. Dies beinhaltet die Datenerhebung und -analyse sowie die Kommunikation und Koordination bei Lagen, die auf übertragbare Krankheiten bezogen sind [2–4].

Das RKI entwickelt Empfehlungen, über deren Umsetzung von Behörden auf lokaler und Landesebene entschieden wird. Im Falle einer epidemisch bedeutsamen Lage kann das BMG dem RKI eine zentrale koordinierende Rolle zuordnen [5].

In diesem Artikel werden die generische Krisenmanagementstruktur des RKI und der Aufbau des RKI-Lagezentrums, deren Operationalisierung im Rahmen der COVID-19-Pandemie und die dabei aufgetretenen Herausforderungen zum Stand 31.10.2020 beschrieben. Ebenso wird der Bund-Länder-Austausch während der bisherigen Pandemie dargestellt.

### Rechtsgrundlagen

Folgende Rechtsgrundlagen definieren die Vorgehensweise und die Verantwortungen hinsichtlich epidemisch bedeutsamer Lagen [1–8]:

- Infektionsschutzgesetz IfSG (2001): §§ 4 und 5 beschreiben die vielfältigen Aufgaben des RKI in gesundheitlichen Krisenlagen,
- Internationale Gesundheitsvorschriften (IGV; 2005),
- Gesetz zur Durchführung der Internationalen Gesundheitsvorschriften (IGV-DG; 2013),
- Beschluss Nr. 1082/2013/EU zu schwerwiegenden grenzüberschreitenden Gesundheitsgefahren (2013),
- Allgemeine Verwaltungsvorschrift über die Koordinierung des Infektionsschutzes in epidemisch bedeutsamen Fällen (IfSGKoordinierungs-VwV; 2013),
- Gesetz zum Schutz der Bevölkerung bei einer epidemischen Lage von nationaler Tragweite (2020),
- Zweites Gesetz zum Schutz der Bevölkerung bei einer epidemischen Lage von nationaler Tragweite (2020),
- Drittes Gesetz zum Schutz der Bevölkerung bei einer epidemischen Lage von nationaler Tragweite (2020).

## Krisenmanagementstruktur des RKI

Das RKI spielt in Deutschland eine zentrale Rolle bei der Bewältigung von Gesundheitsgefahren biologischen Ursprungs. Das Krisenmanagement des RKI hat das Ziel, effektiv zur Bekämpfung der COVID-19-Pandemie beizutragen und die Arbeitsfähigkeit des RKI auch bei hoher Belastung über längere Zeit aufrechtzuerhalten.

Die Grundlage des RKI-Lagemanagements ist der RKI-interne Krisenplan, der darauf abzielt, Arbeitsabläufe innerhalb des RKI transparent darzustellen und dadurch die interne und externe Zusammenarbeit zu verbessern. Er beschreibt das strategische und operative Management, die Organisation, die Verfahren, die Strukturen sowie generische und spezifische Module im Zusammenhang mit Infektionskrankheiten.

Der interne Krisenplan definiert hierfür besondere Strukturen, Abläufe und Zuständigkeiten, die lagebezogen eingesetzt werden können, um flexibel auf Belastungen zu reagieren. Er ist in 3 Stufen unterteilt. Die höchste Stufe, in der der RKI-Krisenstab als strategische und das RKI-Lagezentrum als operative Einrichtung aktiv sind, wurde vor der COVID-19-Pandemie nur 2‑mal aktiviert: bei der Influenzapandemie H1N1 2009/2010 und bei dem EHEC-Ausbruch 2011 (*Escherichia coli*; hämolytisch urämisches Syndrom, HUS; [9, 10]).

**Figure 1:**
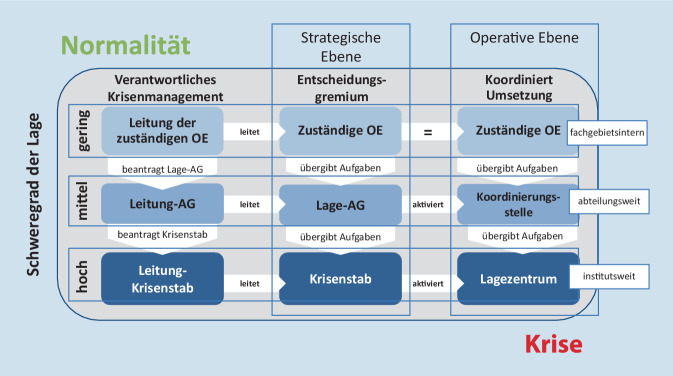

Um über eine mögliche Kriseneskalation zu entscheiden, werden u. a. folgende Indikatoren betrachtet: Anzahl der (möglicherweise) von der Infektionskrankheit betroffenen Personen, Schweregrad, geografische Verteilung, öffentliche Wahrnehmung sowie interne Arbeitsbelastung im RKI. In Abhängigkeit von diesen wird über die Stufe der Eskalation entschieden (Abb. 1).

In epidemisch bedeutsamen Lagen müssen einerseits die für das strategische Management wichtigen Informationen und Daten zielgerichtet und schnell eingeholt und Empfehlungen entsprechend erstellt bzw. angepasst werden. Andererseits müssen die entscheidenden Fakten und Maßnahmen schnell, klar, eindeutig und transparent sowie zielgruppenorientiert nach außen kommuniziert werden. Eine effektive Kommunikation, sowohl von außen ins RKI als auch innerhalb des RKI und aus dem RKI heraus, ist Voraussetzung für ein erfolgreiches Lagemanagement.

**Figure 2:**
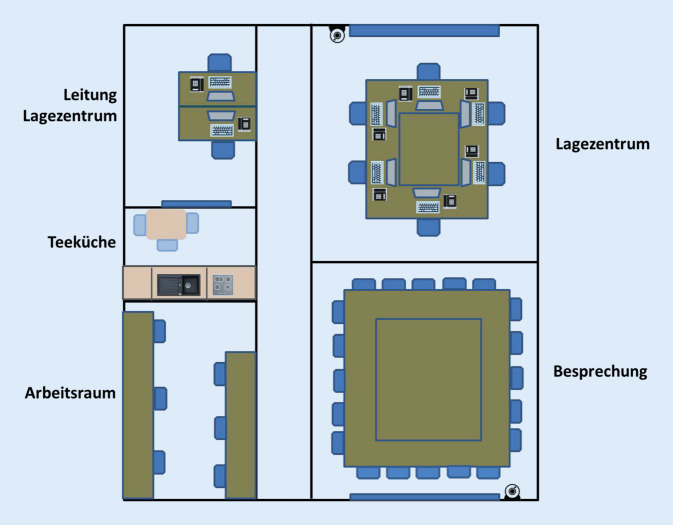

Um epidemisch bedeutsame Lagen auch operativ effizient zu bearbeiten, wurde am RKI ein Lagezentrum eingerichtet. Es hat einen für den Krisenmodus relevanten separaten Stromkreis, erfordert eine zusätzliche Zugangskontrolle und besteht aus den in Abb. 2 dargestellten Räumlichkeiten. Neben der physischen Infrastruktur gibt es definierte Ordnerstrukturen mit Musteranleitungen und Standardarbeitsanweisungen (SOPs) sowie Vorlagen für Situationsberichte, Schichtpläne, Protokolle, Zuständigkeiten usw. Diese wurden bereits bei vergangenen epidemischen Lagen angewandt und fortentwickelt. Mindestens einmal im Jahr wurde eine Schulung zur Arbeit im Lagezentrum durchgeführt, um im Falle einer biologischen Lage einen schnell einsetzbaren Personalpool zu schaffen. Damit das Material sofort nach einer kontextuellen Anpassung genutzt werden kann und um eine Kapazitätenerweiterung (Surge Capacity) zu ermöglichen, wird Software wie Microsoft Word, PowerPoint und Excel verwendet, die weitverbreitet und deren Anwendung meist bekannt ist.

Das RKI ist bei der Bewältigung epidemisch bedeutsamer Lagen ein Akteur unter vielen, hat aber eine zentrale koordinierende Rolle [5]. Deshalb sind eine enge Vernetzung mit anderen Akteuren und eine kontinuierliche Pflege der Kontakte unerlässlich. Die Kommunikation mit der Bevölkerung obliegt in erster Linie der Bundeszentrale für gesundheitliche Aufklärung (BZgA), wenn auch das RKI neben seiner Aufgabe, die Fachöffentlichkeit zu informieren, in epidemisch bedeutsamen Lagen ebenfalls als direkte Informationsquelle für die Bevölkerung genutzt wird, insbesondere durch die Medien. Demnach ist ein guter Austausch der 2 Behörden von großer Wichtigkeit. Das Konzept für die Kommunikation des RKI mit der breiten Fachöffentlichkeit und der Bevölkerung/den Medien wird durch die strategischen Managementstrukturen unter Einbindung der RKI-Pressestelle festgelegt.

## Chronologie der COVID-19-Pandemie

Die das RKI-Lagemanagement betreffenden Eckpunkte der COVID-19-Pandemie sind im Folgenden dargestellt:

- 31.12.2019
  - China meldet dem Landesbüro der Weltgesundheitsorganisation (WHO) in China ein Pneumoniecluster in Wuhan
  - RKI beginnt das Monitoring der Situation
- 06.01.2020 Aktivierung einer institutsweiten Lage-Arbeitsgruppe (AG) am RKI
- 07.01.2020 erste RKI-Risikobewertung wird online gestellt
- 14.01.2020 Aktivierung der Koordinierungsstelle am RKI
- 23.01.2020 erster RKI-Situationsbericht (zunächst nur für Behörden; ab Anfang März auch online verfügbar)
- 27.01.2020 Meldung des ersten laborbestätigten COVID-19-Falles in Deutschland
- 28.01.2020 Aktivierung des Lagezentrums am RKI
- 30.01.2020
  - RKI erhält Mandat für koordinierende Rolle nach § 5 IfSGKoordinierungs-VwV [5], damit übernimmt das RKI zusätzliche Aufgaben, um den Informationsaustausch zwischen Bund und Ländern zu verbessern und die beteiligten Behörden zu Ermittlungen und Maßnahmen zu beraten
  - Entsendung des ersten RKI-Teams zur Unterstützung des Bayerischen Landesamtes für Gesundheit und Lebensmittelsicherheit (LGL) und der in Bayern involvierten Gesundheitsämter beim ersten COVID-19-Cluster in Deutschland [11]
  - WHO erklärt den Ausbruch des neuartigen Coronavirus (2019-nCoV) zur „gesundheitlichen Notlage von internationaler Tragweite“ (Public Health Emergency of International Concern – PHEIC; [12])
- 25.03.2020 Feststellung einer epidemischen Lage von nationaler Tragweite durch den deutschen Bundestag
- 27.03.2020 Gesetz zum Schutz der Bevölkerung bei einer epidemischen Lage von nationaler Tragweite wird verabschiedet [6]
- 19.05.2020 das Zweite Gesetz zum Schutz der Bevölkerung bei einer epidemischen Lage von nationaler Tragweite wird verabschiedet [7]
- 18.11.2020 das Dritte Gesetz zum Schutz der Bevölkerung bei einer epidemischen Lage von nationaler Tragweite wird verabschiedet [8]

## RKI-internes Lagemanagement während der COVID-19-Pandemie

Das RKI hat die COVID-19-Lage seit Beginn der Pandemie beobachtet und bewertet. Zunächst wurde laut RKI-internem Krisenplan die Eskalationsstufe 2 gemäß den Krisenmanagementstrukturen am RKI aktiviert: Am 06.01.2020 wurde eine Lage-AG etabliert, welche folgende 3 Hauptaufgaben hat:

- Lagebewertung,
- strategisches Lagemanagement,
- grundsätzliche Entscheidungen, insbesondere zu Ressourcen und Kommunikation.

Um auch auf operativer Ebene auf den durch die COVID-19-Pandemie erhöhten Kommunikations- und Koordinationsbedarf besser eingehen zu können und um die primär fachlich zuständigen Organisationseinheiten (OE) am Institut zu entlasten, wurde am 14.01.2020 eine Koordinierungsstelle am RKI eingerichtet. Bestehende Ordnerstrukturen sowie diverse Dokumente (Arbeitsanleitungen, Protokolle, Schulungsmaterial usw.) wurden dem Kontext angepasst und angewandt und während der sich weiter entwickelnden Lage regelmäßig überarbeitet. Die Koordinierungsstelle war zunächst mit 2 Positionen besetzt und hat anhand eines dedizierten E‑Mail-Postfachs und einer dedizierten Telefonnummer eingehende Anfragen bearbeitet, organisatorische Vorbereitung und Begleitung von Treffen sichergestellt, wichtige Ereignisse dokumentiert sowie Aufgaben und externe Kommunikation koordiniert.

Mit dem Auftreten des ersten COVID-19-Falles in Deutschland wurde die Lage zu einer epidemisch bedeutsamen Lage mit erhöhtem Schweregrad und infolgedessen die 3. und höchste Eskalationsstufe des RKI-internen Krisenplans aktiviert: Aus der *Lage-AG *wurde der *Krisenstab*, aus der *Koordinierungsstelle* wurde das *Lagezentrum*. Der Krisenstab ist wie zuvor die Lage-AG für das strategische, das Lagezentrum für das operative Lagemanagement verantwortlich (Abb. 1).

Im *Krisenstab* sind neben Vertreterinnen und Vertretern aus verschiedenen OE des RKI auch die BZgA, die Bundeswehr und das BMG bei den Krisenstabssitzungen als Gäste anwesend. Zunächst tagte der Krisenstab täglich von Montag bis Freitag, seit dem 15.06.2020 tagte der Krisenstab 3‑mal pro Woche. Anfangs fanden die Treffen im Besprechungsraum der Koordinierungsstelle bzw. des Lagezentrums statt, seit dem 16.03.2020 werden sie virtuell durchgeführt. Bis zum 31.10.2020 fanden 157 Treffen statt, die in der Regel jeweils 2 Stunden dauerten.

Mit der Überführung der Koordinierungsstelle in das *Lagezentrum* wurden die Anzahl der Positionen und die Anzahl der Mitarbeitenden entsprechend der Arbeitsbelastung wesentlich erhöht. Essenziell war die Rekrutierung von ausreichend Personal aus anderen OE des RKI, um den Betrieb des Lagezentrums aufrechtzuerhalten.

Seit dem 25.01.2020 war zunächst die Koordinierungsstelle, dann auch das Lagezentrum an den Wochenenden besetzt. Die Betriebszeiten waren zunächst von 9–16 Uhr, wurden aber seitdem lage- und bedarfsbedingt angepasst. In Hochzeiten war das Lagezentrum montags bis sonntags von 8–21 Uhr besetzt.

Innerhalb des Lagezentrums wurden im Schichtbetrieb prinzipiell folgende Positionen (Aufgabenbereiche) besetzt (teilweise mehrfach, z. B. Internationale Kommunikation teils mit 12 Personen pro Tag):

- Schichtleitung: Entscheidungstragende der Schicht, Gesamtüberblick und Verantwortung,
- Sichtung: Lesen eingehender E‑Mails und Zuordnung nach Zuständigkeiten,
- Telefon (Sichtung 2): Beantworten des dedizierten Telefons, Unterstützung der Sichtung,
- Aufgaben: Zuordnung und Koordination der eingehenden Aufgaben,
- Lageprotokoll: Führen des Ereignisprotokolls, Dokumentenablage,
- Lagebericht: Erstellung des täglichen Situationsberichts,
- Internationale Kommunikation: Bearbeitung von internationalen Meldungen, Austausch mit anderen Ländern, insbesondere zur Kontaktpersonennachverfolgung,
- Liaison Presse: Beantwortung externer Anfragen die nicht von der Pressestelle bearbeitet werden.

Im RKI wurden außerhalb des Lagezentrums die Positionen „Leitung Lagezentrum“ und „Service“ geführt. Darüber hinaus wurde Anfang März 2020 eine Position für telefonische Anfragen aus der Ärzteschaft eingeführt und Ende April die Position „Kapazitätenmonitoring“, bei der die Gesundheitsämter mit (absehbaren) Problemen bei der Kontaktpersonennachverfolgung behördenintern erfasst werden. Seit Ende August 2020 gibt es die Position Ausbruchsscreening mit dem Ziel eines intensivierten Monitorings von Ausbruchsgeschehen.

Seit dem 23.01.2020 wird vom RKI täglich einen COVID-19-Situationsbericht verfasst. Die Inhalte wurden mit der Zeit angepasst und erweitert. Er beinhaltet u. a. aktuelle Daten zur epidemiologischen Lage in Deutschland und weltweit sowie zu den intensivmedizinischen Behandlungskapazitäten, zudem die aktuelle Risikobewertung, aktualisierte Empfehlungen, Maßnahmen und an festgelegten Wochentagen zusätzliche Informationen zu speziellen Themen (z. B. Testzahlen). Seit dem 01.02.2020 wird der Situationsbericht in leicht verkürzter Form auch auf Englisch veröffentlicht. Zunächst wurden die Berichte nur an Behörden versendet; seit Anfang März 2020 sind diese auf der RKI-Webseite verfügbar [13].

Den Umfang der Lagezentrumsarbeit verdeutlicht die Tab. 1 in Zahlen.

**Table 1:** 

Art	Summe	Kommentar/Zusatz
Tage Koordinierungsstelle/Lagezentrum aktiv	292	–
Kumulative Personenschichten	4504	–
Durchschnittliche Schichten pro Woche	107	(min. 19; max. 167)
E‑Mails im dedizierten Postfach	116.530	–
Einträge in das Lageprotokoll	1638	–
Vergebene Aufgaben	2107	–
Telefonanrufe im Telefonprotokoll	1294	–
Aktivitäten zur Kontaktpersonennachverfolgung durch die Position Internationale Kommunikation	6162	–
Situationsberichte auf Deutsch	283	–
Situationsberichte auf Englisch	274	–

## Bund-Länder-Austausch

Auch außerhalb von epidemisch bedeutsamen Lagen findet ein regelmäßiger Austausch zwischen dem RKI und den verantwortlichen Landesbehörden, Seuchenreferentinnen und Seuchenreferenten und Mitgliedern des Öffentlichen Gesundheitsdiensts (ÖGD) in den Bundesländern statt. Die Fachexpertise aus den Bundesländern fließt über zahlreiche Gremien und Netzwerke durch Diskussionen und Abstimmungen in Dokumente, Veröffentlichungen und Empfehlungen ein.

Bund-Länder-Gremien sind z. B.

- Epidemiologische Lagekonferenz (EpiLag)/Bund-Länder-Arbeitsgruppe (BLAG): Dieses Gremium beinhaltet Vertreterinnen und Vertreter der zuständigen oberen Landesgesundheitsbehörden der 16 Bundesländer sowie der Bundeswehr und des RKI, die sich wöchentlich eng zur epidemiologischen Lage und zu weiteren Themen austauschen.
- Arbeitsgruppe Infektionsschutz (AGI) der Arbeitsgemeinschaft der Obersten Landesgesundheitsbehörden (AOLG): In dieser sind die Seuchenreferentinnen und Seuchenreferenten der zuständigen obersten Landesgesundheitsbehörden der 16 Bundesländer vertreten; Ziel sind die Diskussion und Abstimmung von Empfehlungen unter Moderation des RKI zusammen mit BMG und BZgA.
- ÖGD-Feedbackgruppe: Hier wurden von Länderseite 1–2 Vertreterinnen oder Vertreter aus Gesundheitsämtern benannt; Ziel ist die Verbesserung von RKI-Dokumenten, damit sie im lokalen Gesundheitsamt praxisnah eingesetzt werden können sowie die Qualitätssicherung und Identifizierung von Bedarfen im ÖGD für weitere Informations- und Unterstützungsmaterialien.
- IGV-Flughafengruppe (siehe auch Beitrag von Kleine-Kampmann et al. in diesem Themenheft): Diese AG der Gesundheitsbehörden auf Landes- und Kommunalebene mit Zuständigkeit für IGV-benannte Flughäfen, BMG, Bundesministerium für Verkehr und digitale Infrastruktur (BMVI) und RKI wurde informell eingerichtet und tagt in regelmäßigen Telefonkonferenzen, um einen Erfahrungsaustausch zu ermöglichen und bundesweit möglichst einheitlich vorzugehen. Teils wurden auch Empfehlungen und Stellungnahmen formuliert.

Bei Bedarf wird die Frequenz des Austauschs und der kollaborativen Beratung durch regelmäßige oder situationsbedingt einberufene Telekonferenzen und Berichte angepasst. So auch während der COVID-19-Lage, in der sowohl der Austausch auf Bundesebene mit verschiedenen Ministerien und Behörden (z. B. mit dem BMG, BZgA oder BMVI) als auch der Austausch mit den zuständigen Behörden der Bundesländer erheblich intensiviert wurde. Regelmäßige Telefonkonferenzen fanden bis zu 2‑mal pro Woche statt.

Hinzu kommen zahlreiche Austausche mit der WHO und dem Europäischen Zentrum für die Prävention und die Kontrolle von Krankheiten (ECDC).

Als Beratergremien nutzt das RKI u. a. den Wissenschaftlichen Beirat, den Expertenbeirat Influenza, den Ständigen Arbeitskreis der Kompetenz- und Behandlungszentren für Krankheiten durch hochpathogene Erreger (STAKOB) und eine interdisziplinäre Gruppe externer Experten u. a. aus den Fachgebieten Epidemiologie und Public Health, Ethik, Hygiene, Mikrobiologie, Medizin, Management, Kommunikation und Psychologie.

Um Gesundheitsämter bei der Kontaktpersonennachverfolgung zu unterstützen und die SARS-CoV-2-Ausbreitung möglichst effektiv zu unterbinden, hat das RKI rund 500 sogenannte Containmentscouts ausgebildet [14]. Diese wurden nach einem bestimmten Schlüssel auf die Bundesländer verteilt, um lokal primär dabei zu helfen, Kontaktpersonen schnell und effektiv nachzuverfolgen.

Seit dem 24.04.2020 dokumentiert die Position „Kapazitätenmonitoring“ am RKI für den behördeninternen Gebrauch eingehende Überlastungsanzeigen lokaler Gesundheitsämter, wenn diese die Durchführung der erforderlichen Infektionsschutzmaßnahmen aus Kapazitätsgründen nicht mehr sicherstellen können. Dabei werden Hintergrund und Unterstützungsbedarf geklärt und wenn möglich Unterstützung eingeleitet [15].

## Herausforderungen für das RKI

Auch wenn die COVID-19-Lage das RKI nicht unvorbereitet getroffen hat, gab es im bisherigen Lagemanagement diverse Herausforderungen. Mit der Überführung der Koordinierungsstelle in das Lagezentrum wurden die Positionen und die Anzahl der Mitarbeitenden entsprechend der Arbeitsbelastung wesentlich erweitert. Stets genug geschultes und einsatzbereites Personal über einen langen Zeitraum im Lagezentrum sicherzustellen, ist eine Herausforderung. Aufgrund der seit Januar 2020 anhaltenden Lage, die voraussichtlich noch im Jahr 2021 bestehen bleibt, kommt es regelmäßig zu Lücken im Lagezentrumspersonalschichtplan. Sie entstehen z. B. durch kurzfristige Einsätze zur Unterstützung bei lokalen Behörden oder aber auch durch Krankheitsausfälle und müssen häufig kurzfristig gefüllt werden.

Das Personal für das Lagezentrum wurde aus allen Abteilungen des RKI rekrutiert. Anfangs fanden wöchentliche Einführungen in die Lagezentrumsarbeit statt, sodass ein Pool von ca. 180 Mitarbeitenden (hauptsächlich wissenschaftliche Mitarbeitende, aber auch zahlreiche Verwaltungskräfte) aus 40 Organisationseinheiten entstanden ist. 120 von diesen haben über 10 Schichten geleistet (Stand 31.10.2020). Das Personal musste ggf. eigene Projekte und Arbeiten depriorisieren und sich schnell auf neue Aktivitäten und Verantwortungen einlassen. Die detaillierten SOPs waren hierbei von Vorteil. Während der Lage gibt es regelmäßige, virtuelle positionsspezifische Feedbackrunden, die zum Austausch und zur Mitteilung von Neuerungen dienten, jedoch auch zur Beseitigung von Unsicherheiten und Unklarheiten.

Auch außerhalb des Lagezentrums gab es im RKI viele neue Aufgaben, die verschiedene Mitarbeitende übernommen haben (z. B. Kapazitätenmonitoring, Ausbruchsscreening, telefonische Anfragen aus der Ärzteschaft). Die flexible Anpassung an neue Aufgaben, die schnelle Erstellung von neuen bzw. Anpassung von bestehenden SOPs und die gute Einarbeitung von neuen Mitarbeitenden waren auch hier essenziell.

Technisch war das RKI-Lagezentrum bereits mit Computern, Telefonen, Büromaterialien usw. gut ausgestattet. Dennoch zeigte sich bald, dass die vorhandenen Arbeitsplätze nicht ausreichten; durch die eintretenden Abstandsregelungen wurde die Arbeitsplatzlage noch weiter verschärft. Dank des hohen Maßes an Flexibilität im Haus wurden schnell neue Arbeitsplätze in zusätzlichen Räumen geschaffen.

Durch die jährlichen Schulungen im Lagezentrum waren bereits Strukturen und SOPs erarbeitet worden, die im Laufe der Lage ergänzt werden mussten. Beispielsweise war im ursprünglichen Konzept die Position „Internationale Kommunikation und Kontaktpersonennachverfolgung“ nicht vorgesehen. Dieses Thema gewann während der COVID-19-Pandemie stark an Relevanz, sodass die Notwendigkeit der Einführung einer separaten Position deutlich wurde, die dann in Spitzenzeiten mit 6 Personen pro Schicht besetzt war.

Eine weitere Herausforderung war die gezielte Informationssteuerung. Das Lagezentrum besitzt nur ein E‑Mail-Postfach, auf das alle Mitarbeitenden des Lagezentrums Zugriff haben. Die Anzahl der eingehenden E‑Mails ist hoch (insgesamt 116.530, Stand 31.10.2020). Da sehr viele Mitarbeitende mit demselben Postfach arbeiten, ist es wichtig, standardisierte Regeln zu haben und eine optimale Koordination sicherzustellen. Die Position „Sichtung“ ordnet den E‑Mails deshalb 10 Farbkategorien und ggf. Priorisierungsfähnchen zu.

Zur Unterstützung der Mitarbeitenden im Lagezentrum wurden die o. g. Feedbackrunden sowie Rücksprachen unter den auf den spezifischen Positionen mitarbeitenden Personen gehalten.

Ein Vorteil des Lagezentrums im RKI ist, dass nur mit allgemein bekannter Software gearbeitet wird, sodass neue Mitarbeitende nicht noch zusätzlich den Umgang mit neuen Programmen oder Datenbanken lernen mussten und schnell einsatzbereit waren. Zukünftig könnte es allerdings für einige Dokumente (z. B. den Schichtplan, die Listen der Internationalen Kommunikation) hilfreich sein, auf Datenbanken umzusteigen, um deren Inhalte besser auswerten zu können.

Die epidemische Lage hat viele Fachgebiete im Institut stark in Anspruch genommen. Die Koordination der Aufgaben und die Erstellung und Aktualisierung von Dokumenten sowie die Entwicklung von Empfehlungen und Strategien waren und sind anspruchsvoll. Die Zusammenarbeit funktionierte trotz hoher Arbeitslast und Zeitdrucks durchwegs gut und die operative Koordination über das Lagezentrum (Versand von Aufgaben, Erinnerung an Fristen, Zusammenführen der Dokumente) war eine wichtige Unterstützung für das Lagemanagement im RKI.

## Fazit

Die COVID-19-Pandemie hat in jeder Hinsicht und für viele Sektoren, wie Gesundheitswesen, Politik, Wirtschaft, Bildung und viele andere, außergewöhnliche Umstände mit sich gebracht: Zum Handlungsdruck und dem Wunsch nach sachgemäßer Kommunikation kommt hinzu, dass die Erkenntnisse bezüglich COVID-19 nur allmählich gewonnen werden und der Interpretationsspielraum der Fakten teils intensive Diskussionen zur Folge hat. All dies hat im gesamten ÖGD zu einer enormen Mehrbelastung in jeder Hinsicht geführt, auch im RKI, wo es zu erweiterten Arbeitszeiten, inkl. Wochenendarbeit, kommt. Die Unterstützung der lagebezogenen Aktivitäten durch Mitarbeitende aus den fachlich betroffenen, aber auch aus anderen Organisationseinheiten, die sich schnell in neue Aufgaben einfinden mussten, soll deswegen besonders hervorgehoben werden. Die effiziente Lagebewältigung und die Koordinierung der zahlreichen involvierten Personen stellen große Herausforderungen dar, einerseits aufgrund der Notwendigkeit, stets genügend Personal und Ressourcen zu sichern, andererseits durch die Pflicht einer langfristig angelegten, kohärenten und intensiven Beratung des BMG und anderer involvierter Ressorts.

Unter hohem Druck hat das RKI Empfehlungen, Stellungnahmen und Bewertungen zu den Themen Fallzahlen und Epidemiologie, Meldung, Diagnostik, Teststrategie, allgemeine Infektionsschutzmaßnahmen, Prävention und Management in Einrichtungen des Gesundheitswesens, Kontaktpersonenmanagement, Therapie und Versorgung, Strategie und Krisenpläne, Forschung, internationale Situation sowie Reiseverkehr erstellt. All dies wurde kontinuierlich aktualisiert und den neuen Erkenntnissen und Entwicklungen angepasst.

Die Sinnhaftigkeit vorausschauender Planung wurde durch die gute Vorbereitung des RKI bestätigt. Die letzte Simulationsübung hatte im Dezember 2019 stattgefunden; geschultes Personal, Schulungsunterlagen, Arbeitsanweisungen und die erforderlichen Räumlichkeiten samt Ausstattung konnten unverzüglich der Lage angepasst und eingesetzt werden.

Dessen ungeachtet muss eine strukturierte Evaluation des Krisenmanagements sowohl während („in action“) als auch nach der Pandemie („after action“) erfolgen, damit Lücken entdeckt und geschlossen werden können. Die bisherigen Erfahrungen im COVID-19-Lagemanagement sollen genutzt werden, um Arbeitsabläufe und Strukturen anzupassen.
